# The relationship between the genetic status of the Vrn-1 locus and the size of the root system in bread wheat (Triticum aestivum L.)

**DOI:** 10.18699/VJ21.093

**Published:** 2021-12

**Authors:** O.G. Smirnova, T.A. Pshenichnikova

**Affiliations:** Institute of Cytology and Genetics of the Siberian Branch of the Russian Academy of Sciences, Novosibirsk, Russia; Institute of Cytology and Genetics of the Siberian Branch of the Russian Academy of Sciences, Novosibirsk, Russia

**Keywords:** bread wheat, root system, drought, Vrn-1, flowering dates, мягкая пшеница, корневая система, засуха, Vrn-1, сроки цветения

## Abstract

One of the main ways to fine-tune the adaptive potential of wheat cultivars is to regulate the timing of flowering using the genes of the Vrn-1 locus, which determines the type and rate of development. Recently, with the use of introgression and isogenic lines of bread wheat, it was shown that this locus is involved in the genetic control of root length and weight both under irrigation and drought conditions. It turned out that the VrnA1 gene is associated with a significant decrease in the size of the root system in a winter genotype. The Vrn-A1 gene had the strongest effect on the reduction of the root system in comparison with the homoeoallelic genes Vrn-B1 and Vrn-D1. The aim of this work was to determine whether the allelic composition of the genes at the Vrn-1 locus affects the root size in seven spring cultivars and in two lines of bread wheat differing in flowering time under conditions of normal watering and drought. The research was carried out in a hydroponic greenhouse; drought was created at the tillering stage. In this work, we have shown that early flowering wheat cultivars with the dominant Vrn-A1а allele have more lightweight and shorter roots under normal watering conditions compared to the late flowering carriers of the dominant homoeoalleles Vrn-B1 and Vrn-D1. In drought conditions, the root length decreased insignificantly, but the weight of the roots significantly decreased in all genotypes, with the exception of Diamant 2. It has been hypothesized that the level of the transcription factor VRN-1 at the onset of drought may affect the size of the root system. The large variability in root weight may indicate the participation, in addition to the Vrn-1 locus, of other gene networks in the formation of this trait. Breeders working to develop early maturing varieties should consider the possibility of reducing the root size, especially in arid conditions. A significant increase in the root size of line 821 with introgressions into chromosomes 2A, 2B, and 5A from T. timopheevii indicates the possibility of using congeners as a source of increasing the trait in wheat.

## Abstract (ru)

Одним из главных способов тонкой настройки адаптационного потенциала сортов пшеницы является регулирование сроков цветения с использованием генов локуса Vrn-1, определяющего тип и скорость развития. Исследования, связывающие сроки цветения с формированием корневой системы в нормальных и засушливых условиях, единичны. Недавно с использованием интрогрессированных и изогенных линий пшеницы было показано, что локус Vrn-1 участвует в генетическом контроле длины и массы корней и угла наклона корней в почве как на поливе, так и на засухе. Из трех гомеоаллельных генов – Vrn-A1, Vrn-B1 и Vrn-D1 – ген Vrn A1 наиболее сильно уменьшал размеры корневой системы у озимого генотипа. Целью нашей работы было определить, влияет ли аллельный состав генов локуса Vrn-1 на развитие корневой системы у различающихся по срокам цветения семи яровых сортов и двух линий мягкой пшеницы в условиях нормального полива и засухи. Исследования проведены в условиях гидропонной теплицы, засуха создавалась на стадии кущения. Мы показали, что раннецветущие сорта пшеницы с доминантным аллелем Vrn-A1а в нормальных условиях полива имеют корни меньшей массы и длины по сравнению с поздноцветущими носителями доминантных гомеоаллелей Vrn-B1 и Vrn-D1. На засухе длина корней уменьшалась незначительно, а вот масса корней достоверно снижалась у всех генотипов, за исключением сорта Диамант 2. Мы предположили, что уровень транскрипционного фактора VRN- 1 на момент наступления засухи может оказывать влияние на размер корневой системы. Большой размах изменчивости по массе корней может свидетельствовать об участии, помимо локуса Vrn-1, других генных сетей в формировании этого признака. Селекционерам, работающим над созданием скороспелых сортов, следует учиты- вать возможность уменьшения размеров корневой системы, особенно в засушливых условиях. Значительное увеличение массы корней у линии 821 с интрогрессиями в хромосомы 2А, 2В и 5А от вида T. timopheevii указывает на возможность использования сородичей в качестве источника увеличения размера корней у пшеницы.

## Introduction

Roots are an integral part of the plant organism. Their development begins at the first stages of ontogenesis. The architecture of the root system determines firm rooting of plants, efficient absorption of nutrients and water from the soil, and interaction with the soil biome. A well-developed shallow root system is able to absorb moisture even from small rains, while a longer root system gains access to moisture accumulated in the deeper layers of the soil. These properties of the root system are especially important in condition of droughts, which currently represent the most serious climate threat worldwide (Ahmad et al., 2017; IPCC, 2018). It was shown that the morphological and functional features of the root system are associated with the preservation of the yield under drought conditions (Comas et al., 2013). The accumulation and practical application of genetic knowledge about the formation of the root system in agricultural crops can lead to the second Green Revolution (Den Herder et al., 2010).

In rice and maize, genes responsible for the formation of the root system have been identified and cloned (Uga et al., 2013; Kitomi et al., 2018). Studies on the genetic control of the root system in bread wheat (Triticum aestivum L.) lag significantly behind the studies carried out on rice and maize. So far, in bread wheat, using bi-parental mapping populations and varietal associative mapping panels, QTLs have been localized on the chromosomes of almost all homoeological groups, which indicates a complex genetic control of this trait in wheat (Ehdaie et al., 2016; Lui et al., 2019). Researchers are also looking for genetic diversity for root size in relatives of bread wheat (Feng et al., 2018). It has been shown that the presence of rye introgression in wheat genotypes leads to a significant increase in root weight and an increase in plant productivity under normal and drought conditions (Ehdaie et al., 2003). A similar effect was found in the wheat line Pavon 76 with introgression from Agropyron elongatum to chromosome 7DL (Placido et al., 2013).

The dominant alleles of the Vrn-A1, Vrn-B1, and Vrn-D1 genes located on chromosomes 5A, 5B, and 5D determine the spring type of wheat development (McIntosh et al., 2013). It was recently found that the Vrn-1 locus is involved in the genetic control of root length, root weight, and root angle in wheat and barley (Voss-Fels et al., 2018). We were the first to show the relationship between the flowering time and the size of wheat root system under drought conditions (Pshenichnikova et al., 2020). Using monosomic lines of cv. Saratovskaya 29 for chromosomes 5A, 5B, and 5D, we discovered that the Vrn-A1 gene most strongly affects the decrease in the size of the root system in comparison with other dominant genes of the Vrn-1 locus. The main task of this work was to determine whether the allelic composition of genes at the Vrn-1 locus affects the development of the root system in different cultivars and lines of bread wheat, differing in terms of flowering time under conditions of normal watering and drought.

## Materials and methods

The studies were carried out on bread wheat accessions that are not related by origin and differ in the allelic composition of the Vrn-1 genes. The set of genotypes included spring cultivars Saratovskaya 29 (S29), Novosibirskaya 67 (N67), Yanetskis Probat (YP), Diamant 2 (Dm2), Milturum 553 (M553), Duvanka, Chinese cultivar Chinese Spring (CS), line 821 with introgressions from T. timopheevii Tausch. into chromosomes 2A, 2B, and 5A of S29 (Leonova et al., 2001) and winter synthetic hexaploid wheat line Synthetic 6x (Syn6x) (AABBDD), obtained from the crossing of T. dicoccoides and Ae. tauschii and carrying introgression on chromosome 5D.

To equalize the vegetative period in the set of genotypes, two of them were vernalized at a temperature of +2 °C and a 12-hour light regime: winter Syn6x for 60 days, and the late-ripening cultivar CS for 30 days. Plants were grown in a hydroponic greenhouse of the Institute of Cytology and Genetics of the Siberian Branch of the Russian Academy of Sciences (Center for Shared Use “Laboratory of Artificial Plant Cultivation”) under 12–14-hour artificial light 45,000–50,000 lx, night temperature 18–20 °C and day temperature 24–26 °C. Expanded clay with a particle size of 5 to 15 mm was used as an artificial soil. Knop’s solution served as a nutrient solution. The plants were grown in two identical bathtubs 500 × 100 × 35 cm in size and about 2 m3 in volume. The distance between the plants was 12 cm.

Each genotype was grown under two irrigation regimes during three growing seasons. Before tillering stage, all plants were watered equally twice a day. After the beginning of tillering, two watering regimes were created. In the control variant, the previous irrigation regime was maintained until the end of the season. In the experimental variant, watering was stopped. The moisture level was measured in both baths once a week using a moisture meter. Humidity in the control experiment was 28–30 % throughout the season. Under drought conditions, the humidity gradually decreased and within a month was established at the level of 10–12 %. These experimental conditions simulate changes in soil moisture in the field during spring sowing in the sharply continental climate of Siberia. The flowering date was noted for each plant. The stage of grain waxy ripeness was considered the end of the experiment, after which watering in the control variant was stopped. After the soil dried out, the plants were dug up with the roots, and the aboveground part was cut off. After measuring the length of the roots, they were left in air until completely dry and weighed.

A.B. Shcherban performed molecular analysis of the allelic state of the genes at the Vrn-1 locus in the studied genotypes according to the previously described method (Shcherban et al., 2012).

Phenotypic data obtained during three growing seasons were combined and analyzed by one-way analysis of variance separately for each trait and each irrigation regime. Comparative analysis between groups of genotypes and watering regimes was performed using Student’s t-test. The drought tolerance index was measured as a percentage and was calculated as the ratio of the mean value of the trait under drought to the mean value of the trait under irrigation, multiplied by 100. To study the relationship between three traits (days before flowering, root length and root weight), Pearson correlation analysis was performed. Analyzes were performed using the statistical package STATISTICA 6.

## Results

Molecular analysis of the allelic composition of the Vrn-1 locus in nine wheat genotypes showed the presence of the dominant allele a at the Vrn-A1 locus in cultivars S29, YP, N67, Dm2 and line 821 with introgression from T. timopheevii, created on the basis of cultivar S29 (Table 1). The recessive allele vrn-A1 was found in cultivars M553, Duvanka, CS, and Syn6x. The samples also differed in the allelic state of the Vrn- B1 locus. The dominant allele Vrn-B1c was found in cultivars S29, YP, and line 821, the dominant allele Vrn-B1a was found in cultivars N67, Dm2, M553, and Duvanka, and the recessive allele vrn-B1 was found in CS and Syn6x. All studied wheat samples, with the exception of CS, had the vrn-D1 recessive allele. The CS had a dominant Vrn-D1a allele (see Table 1).

**Table 1.**
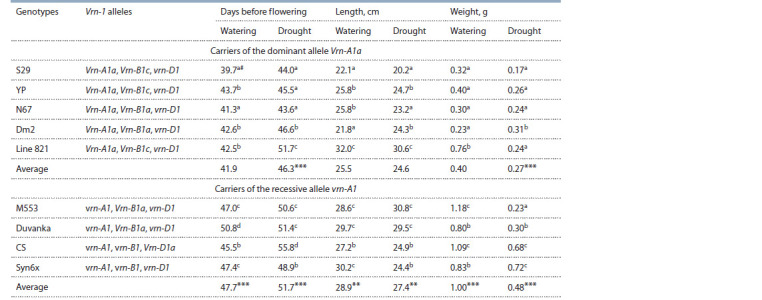
Average values of the number of days before f lowering, length and weight of roots in bread wheat genotypes differing in the allelic composition of the Vrn-1 locus under watering and drought # The mean values followed by different letters in the column are signif icantly different according to the LSD at p = 0.05 within the entire set of genotypes. Differences between the mean values of traits in the groups of carriers of different alleles of the Vrn-A1 gene are signif icant at ** р < 0.01, *** р <0.001.

As a result of the analysis of the allelic state of the Vrn-1 genes, the studied samples were divided into two groups. The first group included four cultivars (S29, N67, Dm2, YP) and line 821 – carriers of the dominant allele of the Vrn-A1a gene. The second group included four cultivars (M553, Duvanka, CS and Syn6x) – carriers of the vrn-A1 recessive allele. Analysis of the flowering time showed that plants in the first group flowered 6 days earlier when irrigated and 7 days earlier in drought than plants in the second group (t = 3.50; p < 0.001). Among the carriers of the Vrn-A1a allele, under both irrigation conditions, cultivars S29 and N67 flowered earlier (see Table 1). Among the carriers of the recessive allele vrn-A1, the earliest cultivars were CS under irrigation and vernalized Syn6x under drought. The average number of days before flowering significantly increased under drought conditions in all studied genotypes (see Table 1). The tolerance index of this trait did not show a large range in the studied genotypes, with the exception of line 821 and cultivar CS, in which the delay in tillering during drought was the most significant (Table 2).

**Table 2.**
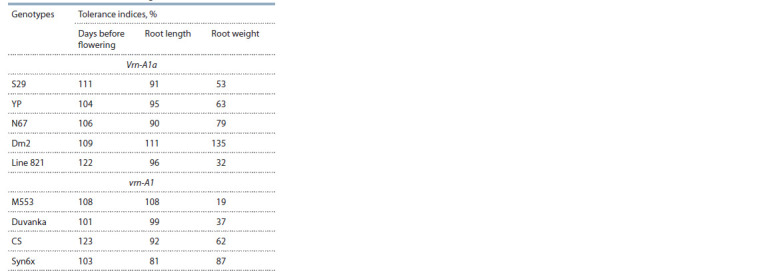
Drought tolerance indices of wheat genotypes with a different allelic state of the genes at the Vrn-1 locus

Much greater variability was shown by root weight, both in absolute values and in the tolerance index to drought. Under normal irrigation, average root weight in the genotypes carrying the dominant Vrn-A1a allele was 0.6 g less than in the carriers of the recessive allele. Under these conditions, four wheat cultivars (S29, YP, N67, and Dm2) from the first group did not differ significantly from each other. The values ranged from 0.23 to 0.40 g (see Table 1). At the same time, the root weight in line 821 belonging to the same group turned out to be almost twice as high as in the cultivars mentioned above. In the group of genotypes – carriers of the recessive allele vrn-A1, cultivars M553 (Vrn-B1a) and CS (Vrn-D1a) had the largest root weight under irrigation, more than 1 g. The weight of roots in cultivars Duvanka and Syn6x was lower, about 0.8 g, and approximately the same as in line 821 from the first group.

Cultivar Dm2 differed from the other genotypes by the increase in root weight during drought. Dm2 showed the highest tolerance index of the trait, 135 % (see Table 2). In other genotypes, the weight of the roots decreased during drought. Cultivar M553 had the most significant 5-fold decrease in root weight with tolerance index 19 %. Cultivar Duvanka, which has the same allelic composition of the Vrn-1 locus as M553, also showed a significant decrease in this trait during drought, tolerance index was 37 %. The same significant decrease in root weight was noted for line 821 from the first group of genotypes. Comparison of tolerance indices among the three studied traits showed that the root weight was the most sensitive to drought in comparison with the root length and the timing of flowering.

Correlation analysis was carried out for three traits for the entire set of genotypes and separately for each of the groups differing in the dominant composition of the Vrn-A1 gene (Table 3). For the entire population, a correlation was found between the number of days before flowering and the root weight, both during irrigation and during drought. The root length correlated with the number of days before flowering only under drought conditions in the entire studied population and among the carriers of the dominant Vrn-A1a allele. The analysis showed that under irrigation conditions there is a correlation between the weight and length of roots for the entire population and for separate groups of genotypes. In drought conditions, this correlation was retained only for the genotypes of the second group with the recessive allele vrn-A1.

**Table 3.**
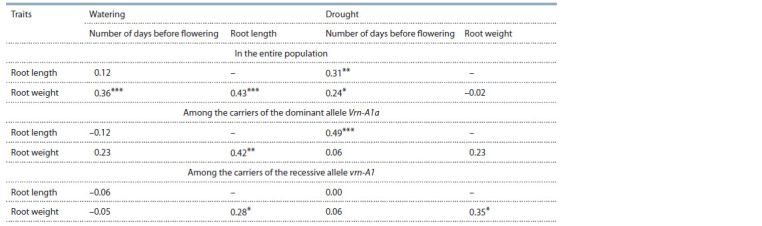
Correlation coeff icients between the number of days before f lowering, root length and root weight under normal watering and drought among nine wheat genotypes differing in the allelic state of the genes at the Vrn-1 locus Notе. Differences are significant at * p <0.05, ** p < 0.01, and *** p < 0.001.

## Discussion

Previously, the involvement of the Vrn-1 locus in the formation of the size of the root system was established, but the study did not use genetic material with the dominant Vrn-A1 allele (Voss-Fels et al., 2018). We have shown that the Vrn-A1 gene, located on chromosome 5A, has the strongest effect on root development compared to the Vrn-B1 and Vrn-D1 genes (Pshenichnikova et al., 2020). This effect was discovered due to the use of a set of S29 monosomic lines. It should be noted that both of these studies were carried out on experimental genetic material using substitution, isogenic or recombinant wheat lines

In this work, using seven spring bread wheat cultivars differing in the time of transition to flowering, and the lines with the introgressions from the tetraploid species T. timopheevii and Ae. tauschii, we estimated the dependence of the development of the root system on the allelic state of the Vrn-1 locus. We also tried to establish how the length and weight of roots in different cultivars are related to the number of days before flowering under normal and drought conditions. The cultivars were divided into two groups. The first group included carriers of the dominant allele Vrn-A1a, the second – carriers of the recessive allele vrn-A1. At the same time, eight out of nine studied genotypes carried dominant alleles of other genes of the Vrn-1 locus

In this work, we have shown that early flowering cultivars with the dominant Vrn-A1a allele had, on average, significantly shorter roots compared to late flowering carriers of the recessive allele. Under irrigation, the difference was 3.4 cm, under drought – 2.8 cm. Cultivars with the recessive allele vrn-A1 also significantly exceeded cultivars of the first group by root weight (see Table 1). Under irrigation, the difference was 0.6 g, and under drought – 0.24 g. Allelism for the Vrn- B1 gene did not affect the size of the root system in carriers of the dominant Vrn-A1a allele.

Great variability in root weight between groups and within groups was found both under irrigation and under drought. The weight increased due to the intensive formation of secondary roots. Most likely, gene networks not associated with the Vrn-1 locus control this process. This assumption was made earlier when the size of the root system was studied in lines with introgression from Ae. tauschii (Pshenichnikova et al., 2020). It was confirmed in the present study when line 821 – the carrier of the dominant Vrn-A1a allele and introgression into chromosomes 2A and 2B – was studied. The length of roots in the line 821 was comparable to the length of roots of the cultivars of the first group (carriers of Vrn-A1a). However, in terms of the weight of roots, line 821 was comparable to the accessions from the second group. Earlier, loci associated with root morphology and size were already identified in chromosomes 2A and 2B in bread wheat (Ehdaie et al., 2016; Liu et al., 2019).

 In our experiment, drought, which occurred at an early stage of plant development, led to an increase in the number of days before flowering in all genotypes (see Table 1). This effect can be considered as the time spent by the plant on the adaptive restructuring of metabolism. Under drought conditions, among all studied genotypes, a correlation was observed between the number of days before flowering and the length and weight of roots. This may indirectly indicate the participation of the Vrn-1 locus in the formation of the root system in response to drought. Under drought conditions, no correlation was observed between the weight and length of roots. Since under irrigation conditions the relationship between these characters was significant( p < 0.001, see Table 3), the lack of correlation under drought conditions may indicate a mismatch in the genetic pathways for the formation of the root system in unfavorable conditions. The correlation analysis carried out for individual groups showed that only among the carriers of the dominant Vrn-A1a allele the number of days before flowering correlated with the length of roots. No correlations were found among the carriers of the vrn-A1 recessive allele. Perhaps this is due to the fact that by the time of the onset and development of drought (the beginning of tillering), the carriers of the dominant Vrn-A1 allele accumulate the transcription factor VRN1 in the leaves in a larger amount than in the carriers of the recessive allele vrn-A1 and the dominant genes Vrn-B1 and Vrn-D1 (Lukoianov et al., 2005). This leads to the interaction of VRN1 with the networks of hormonal and signaling responses at earlier stages and the arrest of root growth. In cultivars that carry only the dominant genes Vrn-B1 and Vrn-D1, this response is delayed, and the roots continue to grow in length.

As has already been noted, the effect of drought on two traits, root length and weight, was different. For each genotype, variability in root length under two irrigation regimes was not significant (see Table 1). This is also evidenced by the drought tolerance indices, which, in general, were close to 100 % (see Table 2). The greatest decrease in root length during drought was observed in vernalized Syn6x. Earlier, we showed that the combined effect of vernalization and drought significantly inhibits the growth of the root system and revealed a weak dependence of the root length under the irrigation conditions (Pshenichnikova et al., 2020). In this work, we have shown that in the studied genotypes the length of the roots depends on the allelic state of the Vrn-1 locus to a greater extent than on the irrigation regime

The weight of roots, in contrast, showed great diversity of variability under drought. This is reflected in the indicators of the tolerance indices (see Table 2). The greatest decrease in the root weight and low tolerance index (53 %) among the cultivars carrying the dominant allele of the Vrn-A1 gene was found in the drought-tolerant cultivar S29. Line 821, which has a genetic basis of cultivar S29, showed the greatest decrease (by 3.2 times) in the weight of roots during drought and the lowest tolerance index in the first group – 32 %. These values are comparable to the decrease in weight in the cultivars from the second group, which form a large root system under favorable irrigation conditions. In Duvanka, the weight of roots decreased 2.7 times with a tolerance index of 37 %, and in M553 – 5 times with the lowest tolerance index – 19 %. Cultivar Dm2 turned out to be the only one of the entire population in which weight of roots and their length were increased. Dm2 was characterized by high tolerance index of the root weight. In general, cultivars, carriers of the dominant Vrn-A1 allele, and vernalized cultivar CS (Vrn-D1 gene) and winter Syn6x had similar dynamics of root weight reduction under drought. Vernalization induces intensive production of the transcription factor VRN-1 (Trevaskis et al., 2007) and thus equalizes the vegetation status of vernalized accessions and cultivars carrying the dominant allele of the Vrn-A1 gene. The level of the transcription factor VRN-1 at the onset of drought may be insufficient for the effective functioning of the gene networks for drought resistance in the non-vernalized cultivars M553 and Duvanka, carriers of the recessive allele of the Vrn- A1 gene and the dominant allele of the Vrn-B1 gene. Thus, the dynamics of the decrease in root weight under drought may indirectly indicate the relationship of the Vrn-1 locus with the gene networks of the response to drought.

The lack of correlation between weight and length of the roots in the entire population under drought indicates the disconnection of the processes of root growth and the accumulation of its biomass through the formation of secondary roots. Each cultivar can use different individual adaptive mechanisms under drought conditions. In particular, cultivar Tincurrin, which has a small root system, has been shown to use soil water more efficiently under drought (Figueroa-Bustos et al., 2020). This was achieved by reducing photosynthetic processes and accelerating grain filling before the onset of serious consequences of water stress. In our experiment, we used cultivar S29, which also has a small root system, but is considered drought-tolerant (Ilyina, 1989). Previously, it was shown that drought resistance of S29 is provided by diverse physiological mechanisms (Osipova et al., 2020).

The mechanisms of the formation of the root system have been studied most fully in the model diploid plant Arabidopsis thaliana. It was found that in Arabidopsis, abscisic acid negatively affected the number and elongation of lateral roots during irrigation (De Smet et al., 2006). Cytokinin has an inhibitory effect on branching of lateral roots, while cytokinin biosynthesis mutants exhibit an increased number of lateral roots (Smith, de Smet, 2012). These and other studies have identified individual components of the development of the root system. At the same time, root development was found to be integrated into systemic signaling that, through sugar metabolism, coordinates whole plant growth during flowering induction (Bouché et al., 2016). Experiments on transgenic barley have shown that the transcription factor VRN1 has, in addition to the main binding sites in the promoters of the flowering initiation genes, secondary binding sites. These sites have been found in genes that play a central role in both hormonal responses and hormone metabolism, which include abscisic acid and cytokinins (Deng et al., 2015). Thus, taking part in the regulation of hormonal pathways, VRN1 can influence the formation of the root system under drought conditions.

## Conclusion

The allelic composition of the Vrn-1 locus determines the time required for wheat plants to enter the generative phase of development. Studies linking the timing of flowering with the formation of the root system under normal and arid conditions are sporadic. In this work, we have shown that cultivars with a dominant allele of the Vrn-A1 gene under normal watering conditions have roots of smaller mass and length compared to carriers of the dominant homoeoalleles Vrn-B1 and Vrn- D1. Drought, which occurs at the tillering stage, led to later flowering of the studied genotypes. At the same time, the length of roots decreased insignificantly, but the weight of roots significantly decreased during drought in all genotypes with the exception of Dm2. The large range of variability in root weight may indicate the participation of additional gene networks in the formation of this trait under drought. Introgressions from T. timopheevii and Ae. tauschii led to an increase in the size of the root system. This indicates the possibility of using congeners as a source of increasing root size in wheat.

Regulation of the flowering time of cultivars in different growing conditions using the Vrn-1 locus is considered one of the main ways to fine-tune the adaptive potential. As our work has shown, one should also take into account the possible relationship of this locus with the size of the root system. Breeders working to develop early maturing cultivars may experience a reduction in the size of the root system, especially in arid conditions. One of the ways to maintain the size of the root system in wheat can be the use of introgressions from species-congeners.

## Conflict of interest

The authors declare no conflict of interest.
